# Diagnosis and management of AL amyloidosis due to B-cell non-Hodgkin lymphoma

**DOI:** 10.3389/fonc.2022.915420

**Published:** 2022-12-15

**Authors:** Callie Berkowitz, Christopher Dittus

**Affiliations:** University of North Carolina at Chapel Hill, Chapel Hill, NC, United States

**Keywords:** IgM AL amyloidosis, lymphoplasmacytic lymphoma/Waldenstrom’s macroglobulinemia, AL amyloidosis, non-Hodgkin lymphoma, IgM paraprotein

## Abstract

Immunoglobulin light chain (AL) amyloidosis may be caused by a B-cell non-Hodgkin lymphoma (NHL) rather than a plasma cell neoplasm in rare cases, which presents unique diagnostic and management considerations. NHL associated with AL will often have an IgM paraprotein; thus, this disease is termed IgM-related AL amyloidosis (IgM AL). The clinical presentation of IgM AL is more likely to involve the lungs, peripheral nerves, and soft tissue; cardiac involvement is less common. Patients with IgM AL amyloidosis should undergo a lymphoma-directed work-up including evaluation for nodal and extranodal disease. Additionally, patients with an IgM paraproteinemia should be screened for AL amyloidosis through history and physical examination. Treatment regimens active against underlying lymphoma, rather than plasma cell-directed regimens, are recommended. Historical response rates in IgM AL have been poor; prospective studies of novel antineoplastic regimens may improve treatment outcomes.

## Introduction

Immunoglobulin light chain (AL) amyloidosis is a rare disease caused by extracellular tissue deposition of protein fibrils. AL amyloidosis is most often associated with an underlying plasma cell neoplasm, but is rarely caused by a B-cell non-Hodgkin lymphoma (NHL). When associated with NHL, there is often an IgM paraprotein present. This is known as IgM-related AL amyloidosis (IgM AL), and represents a distinct clinical entity with unique diagnostic and management considerations. Approximately 5-7% of patients diagnosed with AL amyloidosis will have IgM-associated disease (1). When compared with non-IgM AL, IgM AL more commonly has soft tissue, lung and peripheral nerve involvement (**Table 1**). Importantly, cardiac involvement has been shown to be less common in IgM AL (32-45% IgM AL vs. 70% with non-IgM AL) (2).

**Table 1:** Comparison of AL Amyloidosis and IgM AL amyloidosis (2, 5, 6).

	AL Amyloidosis	IgM AL Amyloidosis
Incidence	10 cases/million person years (1,275-3,200 cases annually in US)	5-7% of AL amyloidosis cases
Median age of diagnosis, years	64	63-68
% Male	65-70%	54-63%
Organ Involvement		
Renal	70%	58%
Cardiac	70%	41%
Peripheral nerve	15%	23%
Hepatic	17%	16%
Soft Tissue	17%	27%
GI Tract	10%	10%
Median NT-ProBNP, pg/ml	2486	1576
Median Troponin-T, ng/ml	0.03	0.01
Mayo Stage at diagnosis		
Stage 1	22%	36%
Stage 2	22%	21%
Stage 3	26%	21%
Stage 4	30%	21%
Treatment Approach	Plasma-Cell Targeted	B-Cell targeted

IgM AL may be associated with many different types of NHL, but it is most commonly associated with lymphoplasmacytic lymphoma/Waldenstrom’s macroglobulinemia (LPL/WM) (74%) (1). An association with other types of NHL is exceedingly rare, and has only been reported in small case series (3). Marginal zone lymphoma, diffuse large B-cell lymphoma, and chronic lymphocytic leukemia have all been associated with both localized and systemic amyloidosis. Extranodal marginal zone lymphoma of mucosa-associated lymphoid tissues (MALT) can be associated with localized amyloid deposition in multiple tissues including skin, conjunctiva, lung, orbit, and gastrointestinal tract (4). While the course is typically indolent in localized amyloidosis, systemic amyloidosis is associated with worse outcomes.

## Clinical presentation & diagnosis

Patients with IgM AL may first present with symptoms related to amyloidosis, IgM monoclonal protein, or lymphoma. In one review, the identification of a monoclonal IgM protein preceded the diagnosis of amyloidosis in about one third of patients (1). Patients with known LPL/WM or other IgM monoclonal gammopathy should be evaluated for possible amyloidosis in certain situations, and patients diagnosed with IgM-associated amyloidosis should complete an additional evaluation for lymphoma (**Table 2**).

**Table 2:** Lymphoma Evaluation for Patients with IgM AL Amyloidosis.

History and Physical Exam	Presence of B-symptoms Examine cervical, supraclavicular, axillary and inguinal lymph nodes Spleen exam
Laboratory Evaluation	Basic labs: CBC, CMP, LDH Monoclonal protein studies: SPEP/SIFE, quantitative immunoglobulins, serum free light chains Beta-2 microglobulin Viral studies: HIV antigen/antibody, HBV serologies, HCV serologies in certain situations Anti-MAG and anti-ganglioside antibodies*
Bone Marrow Evaluation	Flow Cytometry and immunohistochemistry Cytogenetics and FISH MYD88 and CXCR4 testing
Nodal/Extranodal Evaluation	PET/CT (or CT chest, abdomen, pelvis with contrast) Consider lymph node biopsy

*if neuropathy present.

### Evaluation for AL amyloidosis in patients with IgM paraproteinemia or NHL

In patients with a known IgM monoclonal gammopathy, clinicians should evaluate carefully for signs and symptoms of amyloidosis when taking a history and conducting a physical examination. An estimated 7.5% of patients with LPL/WM are also diagnosed with AL amyloidosis at some point in the course of their disease (7), and a very small fraction of patients with IgM monoclonal gammopathy of undetermined significance (MGUS) will develop AL amyloidosis. The clinical presentation of amyloidosis is highly variable and may manifest as abnormalities in many different organ systems including: cardiac (restrictive cardiomyopathy), renal (nephrotic syndrome), hepatic (hepatomegaly), neurologic (peripheral neuropathy, dysautonomia), soft tissue (macroglossia, pseudohypertrophy), and hematologic (abnormal coagulation, bleeding dialysis, easy bruising). Physical exam should include orthostatic vital signs, neurologic examination, cardiopulmonary examination with attention to signs of volume overload, palpation of liver and spleen, examination of the tongue for macroglossia, and skin examination for purpura and ecchymoses. Further laboratory evaluation for end organ damage may include serum BNP/NT-proBNP, urine protein to creatinine ratio, and serum alkaline phosphatase if clinical signs or symptoms of cardiac, renal, or hepatic involvement are present (2).

Neuropathy can pose a diagnostic dilemma, as this symptom is common in the general population and can be seen both in IgM monoclonal gammopathies as well as AL amyloidosis. Patients with neuropathy should undergo screening for other common etiologies such as diabetes, B12 deficiency, and alcohol use. Further evaluation includes nerve conduction studies, electromyography (EMG) and testing for anti-myelin associated antibodies (MAG) and anti-ganglioside antibodies. Monoclonal gammopathy of neurologic significance (MGNS) may be diagnosed if the following factors are present: symmetric sensory deficits, slow progression, length dependence, strongly positive anti-MAG antibodies, and prominent demyelination on EMG. Features less consistent with MGNS (asymmetry, motor-predominant, rapidly progressive, length independent, negative MAG-antibodies, or axonal nerve conduction) should prompt a work-up for other diseases that may be associated with an IgM paraprotein and peripheral neuropathy, such as AL amyloidosis, POEMS (polyneuropathy, organomegaly, endocrinopathy, monoclonal protein, skin changes), cryoglobulinemia, and Bing-Neel syndrome (8).

The majority of patients will not require amyloid-specific evaluation beyond the history and physical exam. However, if suggestive symptoms are present, evaluation for potential amyloidosis should be pursued as detailed in the relevant review chapter. Of note, IgM MGUS can co-exist with other forms of systemic amyloidosis; presence of AL amyloidosis must be definitely determined in a biopsy specimen with mass spectrometry or immune electron microscopy (2).

### Evaluation for lymphoma in patients with IgM AL

In addition to routine amyloidosis evaluation and staging, patients diagnosed with IgM AL should undergo an additional lymphoma evaluation. This differs significantly from patients with non-IgM AL who will undergo a workup for underlying plasma cell neoplasm. The initial evaluation includes the history and physical exam. Patients should be asked about the presence of B-symptoms, including night sweats, fever, or persistent weight loss. On physical exam, patients with IgM AL should have a thorough lymph node exam with particular attention paid to the cervical, supraclavicular, axillary and inguinal lymph nodes; the spleen should also be examined. Laboratory evaluation should include complete blood count (CBC), comprehensive metabolic panel (CMP), serum protein electrophoresis with immunofixation (SPEP/SIFE), quantitative immunoglobulins, serum free light chains, beta-2 microglobulin, lactate dehydrogenase (LDH), human immunodeficiency virus (HIV) antigen/antibody. Hepatitis B virus (HBV) serologies should be obtained given risk of HBV reactivation with rituximab therapy. Hepatitis C virus (HCV) serologies should be sent if there is a concern for splenic marginal zone lymphoma.

The workup for AL amyloidosis, as well as LPL/WM, includes a bone marrow biopsy. Bone marrow samples should be sent for MYD88 testing and consideration of CXCR4 testing in addition to flow cytometry, immunohistochemical staining, and cytogenetic analysis. MYD88 gene mutation is present in over 90% of patients with LPL/WM and may helpful when the diagnosis is uncertain (9). CXCR4 is present in 30-40% of patients with LPL/WM and may be associated with resistance to ibrutinib (9). In one single institution study of 75 patients with IgM AL, 58% of patients had a positive MYD88 and 17% positive CXCR4 (17%) (6).

Imaging should be obtained to evaluate for sites of NHL involvement, particularly the lymph nodes. Extranodal involvement may be present, but classic osteolytic bone lesions, as found in multiple myeloma, are less of a concern. Positron emission tomography/computerized tomography (PET/CT) is preferred for NHL staging evaluation, but computed tomography (CT) of the chest, abdomen, and pelvis with intravenous contrast can be a reasonable alternative in certain clinical situations (9). If lymphadenopathy is present, excisional lymph node biopsy may be obtained, especially if there is diagnostic uncertainty following the bone marrow biopsy. Central nervous system involvement is rare in LPL/WM (described as Bing Neel syndrome); MRI and LP is only obtained when suggestive symptoms are present.

## Prognosis

Most prognostic evaluations of AL amyloidosis involve measures of cardiac dysfunction such as cardiac troponin or NT-proBNP. As cardiac involvement is less common in IgM AL, patients generally present with lower stage disease compared to non-IgM AL. Median survival of IgM AL is 49-78 months, similar to non-IgM AL (2). However, patients with early stage (stage I or II) IgM AL have poorer survival compared with early stage patients with non-IgM AL (75% OS at 5 yrs for stage 1 IgM AL vs. >90% non-IgM AL) and have lower treatment response rates (1, 6). In turn, developing efficacious treatments in this rare subtype of an already rare disease is challenging, with treatment decisions relying primarily on retrospective data.

Patients with LPL/WM and amyloidosis have significantly worse overall survival than patients with LPL/WM alone (2.5 years vs. 12.1 years; HR 5.9) (7). The best overall survival rates occur in IgM AL when no clonal infiltrate is present in the bone marrow versus a lymphoid infiltrate versus a plasma cell infiltrate (54 months vs. 44 months vs 23 months in case series) (1). Negative prognostic factors include advanced age (>66), presence of neuropathy, liver or cardiac involvement, multiorgan involvement, and low albumin (<3 g/dL) (1, 2). Response to treatment is an important predictor of survival, with most patients achieving a partial response (32-74%) and fewer achieving very good partial response or complete response (2). While further research into treatment approaches may lead to improved outcomes in this population, prospective studies are challenging to conduct in this rare disease. As treatment paradigms shift, ongoing survival assessments are needed.

## Treatment

The overall goal of treatment is to eliminate the production of amyloid protein by targeting the underlying B-cell NHL. Typical plasma cell regimens are less effective in IgM AL, as they do not target the appropriate underlying clonal disorder (10). Patients should be referred to an amyloidosis center for multidisciplinary care, with input from both amyloidosis, lymphoma, and bone marrow transplant specialists in determining an optimal treatment plan. Based on amyloid organ involvement, patients may require additional subspecialty consultation including cardiology, nephrology, and hepatology. Due to the rare nature of this disorder, clinical trial data remains limited, with most recommendations based on small non-randomized studies and expert opinion.

While IgM AL has historically been treated with conventional chemotherapy alone (e.g. melphalan, cyclophosphamide), rituximab-based regimens along with bortezomib, immunomodulatory imide drugs (IMiDs), and Bruton’s tyrosine kinase inhibitors (BTKi) have been introduced into clinical practice and evaluated primarily in the form of small retrospective analyses and case series (1, 11, 12). Toxicity profiles and underlying hematopathology are important in selecting treatment. Treatment regimens with efficacy for LPL/WM that have been studied in IgM AL include bendamustine in combination with rituximab (BR), bortezomib, dexamethasone, and rituximab (BDR), and the BTKi ibrutinib. Daratumumab has been studied in LPL/WM, but was not found to have high response rates (13).

In a small retrospective study with 27 patients, BR showed an overall response rate of 59%, with 11% of patients achieving complete response (14). BR is not associated with neurologic, renal, or cardiac side effects, and thus may have a favorable toxicity profile in patients with AL amyloidosis. Importantly, though, bendamustine has renal excretion and dosing may be affected by patients with renal failure. BDR was studied in a 10-patient single-arm prospective study, and resulted in a partial response rate of 78% (15). However, neuropathy related to bortezomib may be a concern in patients with baseline neuropathy from amyloidosis.

In a retrospective study of ibrutinib in 8 patients with IgM AL, only 2 (25%) achieved a hematologic response. Peripheral edema was described in 4 of 8 patients and atrial fibrillation in 2 of 8 patients (16). A subsequent retrospective analysis of 4 patients with IgM AL treated with BTKi therapy (ibrutinib or acalabrutinib) as monotherapy or combined with rituximab showed favorable hematologic response (VGPR in 2 patients and CR in 2 patients) (12). Ibrutinib may be more challenging to tolerate in patients with amyloidosis due to the toxicity profile (bleeding and arrhythmias); while more selective BTKi agents, such as acalabrutinib or zanubrutinib, may be better tolerated. Zanubrutinib has been compared to ibrutinib in a randomized trial for the treatment of LPL/WM and was associated with similar efficacy, but lower rates of bleeding and atrial fibrillation (16).

Appropriately selected patients may be referred for autologous stem cell transplant. In a retrospective analysis of 38 patients who underwent transplant for IgM AL, response rates were high (92% overall response) (17). Over half of patients (58%) had received at least 1 prior line of therapy. Median progression free and overall survival were 48 and 106 months, respectively. However, 2/38 (5%) died within 100 days of transplant (17). In a review of 250 patients with IgM AL at European amyloidosis centers, only 4 patients (2%) underwent transplant; all patients were alive 2 years post-transplant (1). Transplant eligibility is dependent on age, patient performance status, co-morbidities, end-organ function, and caregiver/psychosocial support.

While no standard treatment guideline exists, a reasonable approach may include BR as frontline treatment and a second generation BTKi at relapse. Determining how to best integrate and sequence autologous stem cell transplant remains a challenge due to limited data, with practices differing between centers. As we are treating an underlying lymphoma, induction treatment should be given prior to transplant consideration. Additionally, patients should be evaluated at specialty centers experienced with transplanting amyloid patients. However, less than a quarter of patients with IgM AL are transplant eligible at presentation (17).

## Conclusion

While uncommon, AL amyloidosis with underlying B-cell NHL is important to recognize due to its unique diagnostic and treatment considerations. Patients with IgM AL amyloidosis should be evaluated for presence of an underlying lymphoma. Additionally, patients with an IgM paraproteinemia should be screened for AL amyloidosis through history and physical examination. Treatment regimens active against underlying lymphoma are recommended, along with consideration of autologous stem cell transplant in appropriately selected patients. Historical response rates in IgM AL have been poor; future prospective studies of modern chemotherapy regimens may improve treatment outcomes.

## Author contributions

CB and CD both contributed to literature review, research analysis, and manuscript drafting. Both authors contributed to the article and approved the submitted version.
